# Development of the stria vascularis in the common marmoset, a primate model

**DOI:** 10.1038/s41598-022-24380-6

**Published:** 2022-11-17

**Authors:** Makoto Hosoya, Tsubasa Kitama, Kaho Iwabu, Takanori Nishiyama, Naoki Oishi, Hideyuki Okano, Hiroyuki Ozawa

**Affiliations:** 1 Department of Otorhinolaryngology, Head and Neck Surgery, Keio University School of Medicine, 35 Shinanomachi, Shinjuku-Ku, Tokyo, 160-8582 Japan; 2 Department of Physiology, Keio University School of Medicine, 35 Shinanomachi, Shinjuku-Ku, Tokyo, 160-8582 Japan; 3 Laboratory for Marmoset Neural Architecture, Center for Brain Science, RIKEN, 2-1 Hirosawa, Wako, Saitama, 351-0193 Japan

**Keywords:** Developmental biology, Neuroscience

## Abstract

Stria vascularis is a structure that generates potassium gradients in the cochlea, which is vital for hair cells to convert mechanical sound waves into electrical pulses. The precise development of the stria vascularis and subsequent generation of endocochlear potential are thus essential for hearing. Understanding the development of the stria vascularis is valuable for studying hearing loss caused by aging or genetics and designing regenerative therapy. Although inter-species differences have been reported between rodents and humans, most of our current knowledge regarding cochlear development has been obtained from rodent models because of the difficulty in using human fetal samples in this field of research. Therefore, we investigated the development of the cochlear stria vascularis in the common marmoset (*Callithrix jacchus*), a small monkey species native to the New World. Our study confirms that stria vascularis development in the common marmoset is similar to that in humans and is suitable for furthering our understanding of human cochlear development. The time course established in this report will aid in studying the primate-specific developmental biology of the inner ear, which could eventually lead to new treatment strategies for hearing loss in humans.

## Introduction

Hearing is the process by which mechanical sound waves are perceived by the inner ear, converted into electrical impulses in the neurons, and recognized by the brain. The inner ear is the peripheral sensory organ responsible for hearing and balance. In mammals, the inner ear is divided into two parts: the cochlea, which is responsible for hearing, and the vestibule and semicircular canals, which are responsible for balance and acceleration. In the cochlea, hair cells convert the mechanical sensation perceived due to sound into neuroelectric pulses. These pulses eventually reach the auditory cortex of the brain, where sound stimuli can be perceived by auditory neurons. This intrinsic generation of electrical pulses in hair cells is driven by a concentration gradient of potassium ions between the hair cells and the endolymph that faces the hair cells.

Most of our knowledge of cochlear development has been obtained from rodent models. Little is known about cochlear development in humans because this process occurs in a relatively late phase of gestation^1–3^, and ethical concerns prevent the study of late-stage fetus samples. Furthermore, our knowledge of cochlear development in rodent models cannot always be directly applied to humans because several inter-species differences in cochlear development in rodents and humans have been reported^1^. To overcome this limitation in sampling and to minimize species differences, the common marmoset (*Callithrix jacchus*), a small primate model, has been used in this study to understand cochlear development^4–6^, as well as genetic or age-related hearing loss^7^. A similar approach has been adopted in research on the central nervous system^8^. We have previously reported on cochlear development in the common marmoset, including its basic anatomical staging compared to humans and mice, as well as the expression patterns observed in conventional markers of hair cells, supporting cells, spiral ganglion neurons, and other cochlear cells^4–6^. Furthermore, we revealed several differences in the development of hair cells and spiral ganglion neurons between rodents and marmosets, and demonstrated the similarity to the development process in humans^4,5^.

The stria vascularis of the cochlea is the primary tissue responsible for generating the concentration gradient of potassium ions essential for hearing^9,10^. Therefore, the fine-tuned development of the stria vascularis in the cochlea is vital for normal hearing^11^. This stria vascularis comprises mainly three layers of cells: the marginal, intermediate, and basal cells^12^. Previous reports reveal that the three layers of the stria vascularis have different origins: the marginal cells are derived from epithelial cells of the cochlear duct that develop from the otocyst, intermediate cells originate from immigrating neural crest cells and Schwann cell precursors, and basal cells differentiate from periotic mesenchymal cells^13–19^. In addition to these three layers, the stria vascularis contains capillaries that directly influence the composition of the endolymph^12,20^.

We lack investigations on the development of the stria vascularis in non-human primates. Such an examination would reveal inter-species differences in the development of the stria vascularis or what has not been previously studied in rodents. Moreover, knowledge of primate development would be useful in regenerative studies in the human stria vascularis. Here, we report a detailed description of cochlear development in *C. jacchus*. In addition, as in humans, cochlear development is slower in this primate than in rodents^1,4^. Therefore, investigating this model animal is thought to be more suitable for detecting brief or transient developmental changes in gene expression patterns.

## Materials and methods

### Specimens

Cadaverous temporal bone samples of common marmosets at E87 (n = 3), E96 (n = 3), E101 (n = 3), E109 (n = 3), E115 (n = 3), and P0 (n = 3) were used in this study. The animal experiments were approved by the Animal Experiment Committee of Keio University (number: 11006, 08020) and were performed following the guidelines of the National Institutes of Health and the Ministry of Education, Culture, Sports, Science, and Technology of Japan.

### Tissue preparation

The temporal bone was dissected immediately after euthanization and fixed with 4% paraformaldehyde in phosphate-buffered saline (PBS) overnight. Specimens were embedded in the Tissue-Tek O.C.T. compound (Sakura Finetechnical Co., Ltd., Tokyo, Japan) for cross-sectioning. P0 specimens were decalcified in Decalcifying Solution B (Wako, Osaka, Japan) for 1 week and then embedded in Tissue-Tek O.C.T. compound for cross-sectioning. Seven-micrometer sections were used for immunohistochemical analysis.

### Antibodies

The following primary antibodies were used: Anti-ATP1A1 (Mouse IgG2a, a6F, DSHB, Iowa City, IA, USA, 1:500), Anti-BSND (Rabbit IgG, ab196017, Abcam, Cambridge, UK, 1:500), Anti-SLC12A2 (Goat IgG, sc-21545, Santa Cruz Biotechnology, Santa Cruz, CA, USA 1:1000), Anti-ATP1B1 (Mouse IgG2a, ab2873, Abcam, Cambridge, UK 1:1000), Anti-MLANA (Rabbit IgG, NBP1-30151, Novus, St. Charies, MO, USA 1:500), Anti-KCNJ10 (Rabbit IgG, APC-035, alomone Labs, Jerusalem, Israel, 1:500), Anti-GLUT1 (Rabbit IgG, ab115730, Abcam, Cambridge, UK, 1:500), Anti-ACTA2 (Mouse IgG2a, A2547, SIGMA, Saint Louis, MO, USA, 1:200), Anti-CD34 (Rabbit IgG, ab81289, Abcam, Cambridge, UK, 1:200), Anti-CLDN11 (Rabbit IgG, ab53041, Abcam, Cambridge, UK, 1:200), Anti-CLDN11 (Mouse IgA, sc-271231, Santa Cruz Biotechnology, Santa Cruz, CA, USA, 1:100).

The following secondary antibodies were used: donkey anti-mouse IgG, Alexa Fluor Plus 488 (A32766, 1:500, Invitrogen), donkey anti-rabbit IgG, Alexa Fluor Plus 555 (A32794, 1:500, Invitrogen), and donkey anti-goat Alexa Fluor 647 (703-605-147 1:500, Jackson Immuno-Research), goat anti-mouse IgG, IgM, IgA, Alexa Fluor 488 (A10667, 1:500, Invitrogen), goat anti-rabbit IgG (A32732, 1:500, Invitrogen).

### Immunohistochemistry

After a brief wash with PBS, the sections were heated (80 °C) in 10 µM citrate buffer (pH 6) for 15 min. After another brief wash, the sections were pre-blocked for 1 h at room temperature in PBS containing 10% normal serum, incubated overnight with the relevant primary antibodies at 4 °C, and then incubated with Alexa Fluor-conjugated secondary antibodies for 60 min at room temperature. Nuclei were counterstained with Hoechst 33,258. We avoided heating the sections for CLDN11 (ab53041) staining.

### Ethics approval and consent to participate

The animal experiments were approved by the Animal Experiment Committee of Keio University (approval number: 11006, 08020) and were performed in accordance with the guidelines of the National Institutes of Health and the Ministry of Education, Culture, Sports, Science, and Technology of Japan.

## Results

### Specification and development of marginal cells of the stria vascularis in the primate

**Figure 1:**
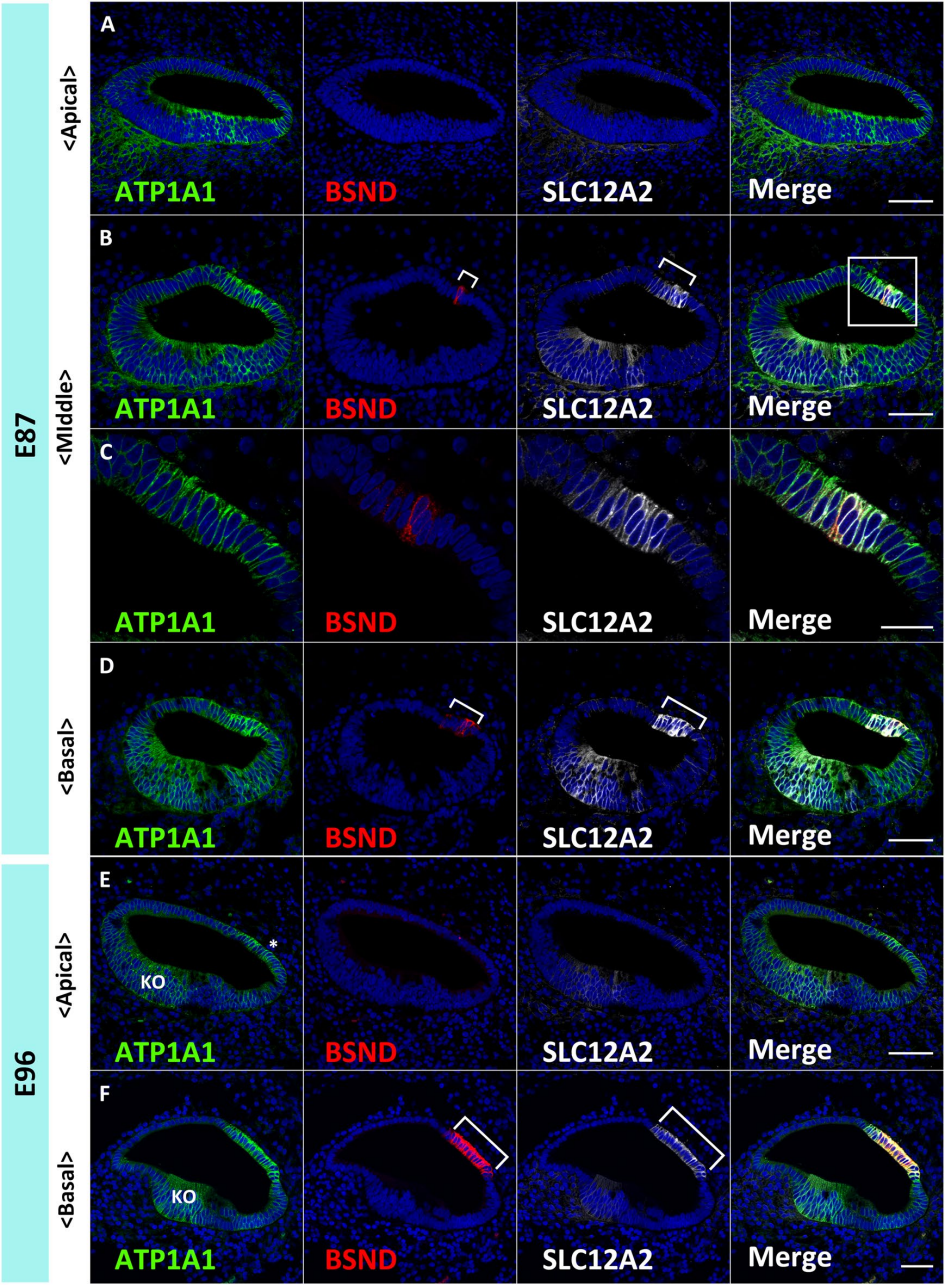
Specifications of the marginal cells of the stria vascularis in the primate cochlea. (**A**–**D**) Expression of ATP1A1, BSND, and SLC12A2 in E87 cochlea. In the apical turn, no BSND or SLC12A2 expression was detected, whereas ATP1A1 expression can generally be observed in the cochlear duct (**A**). In the middle turn, BSND expression can be detected as a narrow band in the lateral wall of the cochlear duct. SLC12A2 expression is also observed in developing marginal cells. SLC12A2 expression in the lateral wall of the epithelium of the cochlear duct is broader than that of BSND (Bracket in **B**) (**C** shows a higher magnification image of the box in **B**) (**B** and **C**). In the basal turn, both BSND and SLC12A2 exhibit broader expression than that observed in the middle turn, whereas BSND expression is narrower than that of SLC12A2 (Bracket in **D**). (**E** and **F**) Expressions of ATP1A1, BSND, and SLC12A2 in the E96 cochlea. In the apical turn, BSND and SLC12A2 expression cannot be detected. ATP1A1 expression is still generally observed in the cochlear duct, although it is more prominent in the lateral wall (* in **E**) and Kölliker’s organ (**E**). In the basal turn, expression of BSND and SLC12A2 is observed in developing marginal cells. Compared to that in the basal turns of E86, their expressions are wider. SLC12A2 expression is still broader than that of BSND (Bracket in **F**). ATP1A1 expression is restricted in developing marginal cells and Kölliker’s organ (**F**). The nuclei were counterstained with Hoechst (blue). Scale bar: 50 µm in **A**, **B**, **D**–**F**, 20 µm in C. KO: Kölliker’s organ.

**Figure 2:**
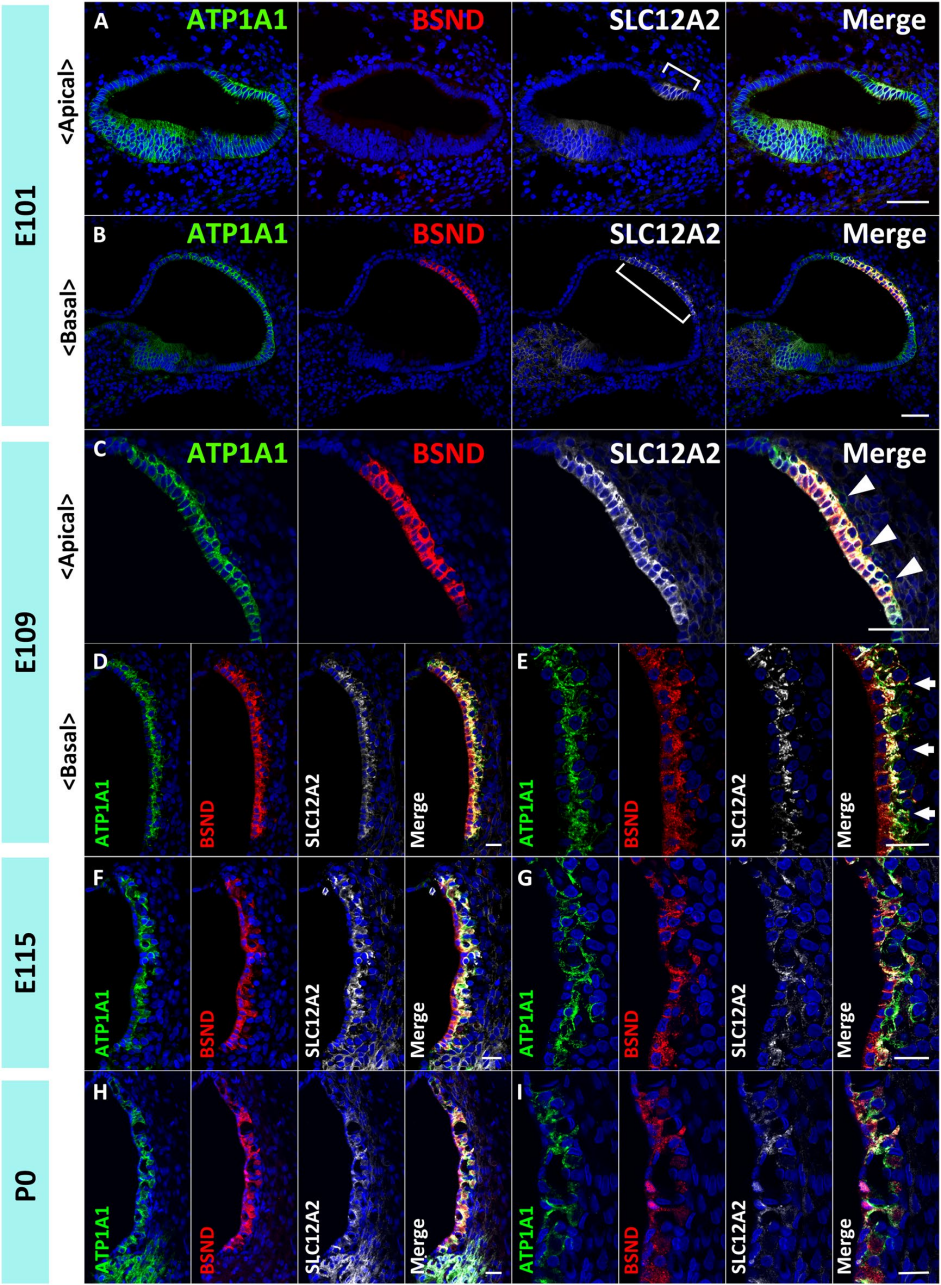
Development of the marginal cells of the stria vascularis in the primate cochlea. (**A** and **B**) Expression of ATP1A1, BSND, and SLC12A2 in E101 cochlea. In the apical turn, no BSND expression is detected, whereas SLC12A2 expression can be observed (Bracket in **A**). In the basal turn, BSND expression can be detected along with SLC12A2 expression. ATP1A1 expression in the lateral wall of the epithelium of the cochlear duct is still broader than that of SLC12A2 (Bracket in **B**). (**C**–**E**) Expression of ATP1A1, BSND, and SLC12A2 in E109 cochlea. At E109, the BSND expression can be observed in the apical turns. The basolateral membrane of the marginal cells is relatively smooth (Arrowheads in **C**). In the basal turns of E109 cochlea, ATP1A1, BSND, and SLC12A2 are expressed in marginal cells. The basolateral membrane of the marginal cells is becoming more intricate compared to the apical turn (Arrows in **E**). (**F** and **G**) Expression of ATP1A1, BSND, and SLC12A2 in E115 cochlea. Expression of ATP1A1, BSND, and SLC12A2 are detected in marginal cells at this stage. The basolateral membrane of the marginal cells has been getting more complex from E109. (**H** and **I**) Expression of ATP1A1, BSND, and SLC12A2 in P0 cochlea. Expression patterns of ATP1A1, BSND, and SLC12A2 are similar to those at E115; hence this stage is morphologically similar to E115. The nuclei were counterstained with Hoechst (blue). Scale bar: 50 µm in **A**–**C**, 20 µm in **D**–**I**.

**Figure 3:**
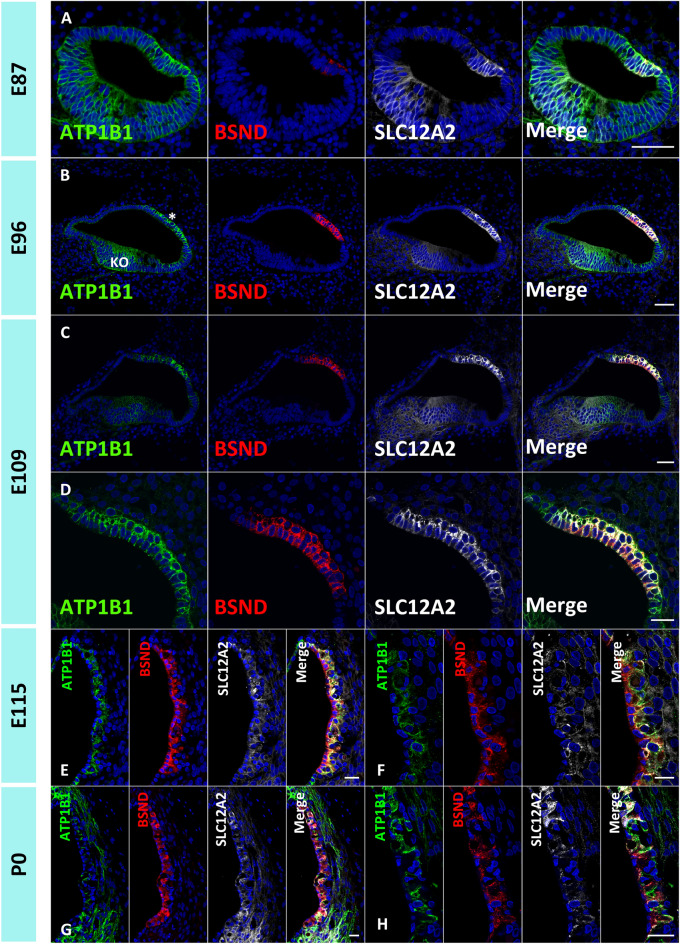
ATP1B1 expression patterns in the marginal cells of the stria vascularis in the primate cochlea. (**A**) ATP1B1 is expressed in the cochlear duct broadly in the E87 cochlea. (**B**) ATP1B1 expression is relatively higher in developing marginal cells (* in B) and Kölliker’s organ at E96, diminishing from other parts of the cochlear duct. (**C** and **D**) ATP1B1 expression is observed in developing marginal cells at E109. (**E**–**H**) At E115 and P0 cochlea, ATP1B1 expression is observed in marginal cells. The nuclei were counterstained with Hoechst (blue). Scale bar: 50 µm in **A**–**C**, 20 µm in **D**–**H**. KO: Kölliker’s organ.

Firstly, in this study, we examined regional specifications and several markers of the development of marginal cells (Figs. 1, 2, 3), barttin CLCNK type accessory subunit beta (BSND), solute carrier family 12 member 2 (SLC12A2), ATPase Na^+^/K^+^ transporting subunit alpha 1 (ATP1A1), and APT1B1, whose expression has been reported in the stria vascularis of rodents^21–23^.

*BSND* encodes an essential beta subunit for chloride channels (CLC) localized to the basolateral membranes of renal tubules and the stria vascularis^22^. Mutations in this gene have been associated with Bartter syndrome and sensorineural deafness^24^. *SLC12A2* (Solute Carrier Family 12 Member 2) encodes a Na^+^/K^+^/2Cl^−^ co-transporter (NKCC1), which is essential for normal hearing^23,25^. *ATP1A1* and *ATP1B1* encode Na^+^/K^+^-transporting ATPase subunit alpha-1 and beta-1^21,26^, responsible for establishing and maintaining the electrochemical gradients of Na and K ions across the plasma membrane. The corroborative activities of proteins in the basal membrane of marginal cells in the stria vascularis are essential for K^+^ circulation and the formation of endocochlear potential in the lateral wall^27^.

First, we examined the E87 cochlea, in which hair cell differentiation has been observed in the sensory epithelium of the basal turns and not in the apical turns^6^. In the E87 cochlea, ATP1A1 expression was observed throughout the cochlear epithelium. In contrast, BSND and SLC12A2 expression was observed only in the basal and middle turns of the cochlear duct, and no expression was observed in the apical turns (Fig. 1A–E). SLC12A2 expression was also observed in the ventromedial regions of the cochlear duct at this stage, whereas BSND expression was only observed in developing marginal cells. Notably, BSND and SLC12A2 expressions were detected in the middle and basal turns; however, the expression of SLC12A2 was significantly broader than that of BSND (Fig. 1, Bracket in B and D).

At E96, BSND and SLC12A2 expression were not observed in the apical turns (Fig. 1E). In contrast, broader expression of BSND and SLC12A2 expression compared to that in E87 was observed in the basal turns (Fig. 1F). At this stage, ATP1A1 expression was restricted to developing marginal cells in the basal turns and a part of Kölliker’s organ. In contrast, a broader expression of ATP1A1 was still observed in the apical turns.

At E101, SLC12A2 expression was observed in the marginal cells from basal to apical turns (Fig. 2A,B), with the expression narrower in the apical turns than in the basal turns. BSND expression could still not be observed in apical turns (Fig. 2A). At this stage, ATP1A1 expression was also restricted to apical turns, as observed in basal turns in the previous step.

At E109, BSND expression was also observed in apical turns, but its expression in developing marginal cells was narrower than that of SLC12A2 and ATP1A1 (Fig. 2C–E). At this stage, the basolateral membrane of marginal cells in the basal turn becomes more intricate than its relatively smooth expression in the apical turn. At E115, expression patterns of BSND, ATP1A1, and SLC12A2 in marginal cells were similar to those at P0 (Fig. 2F–I).

**Figure 4:**
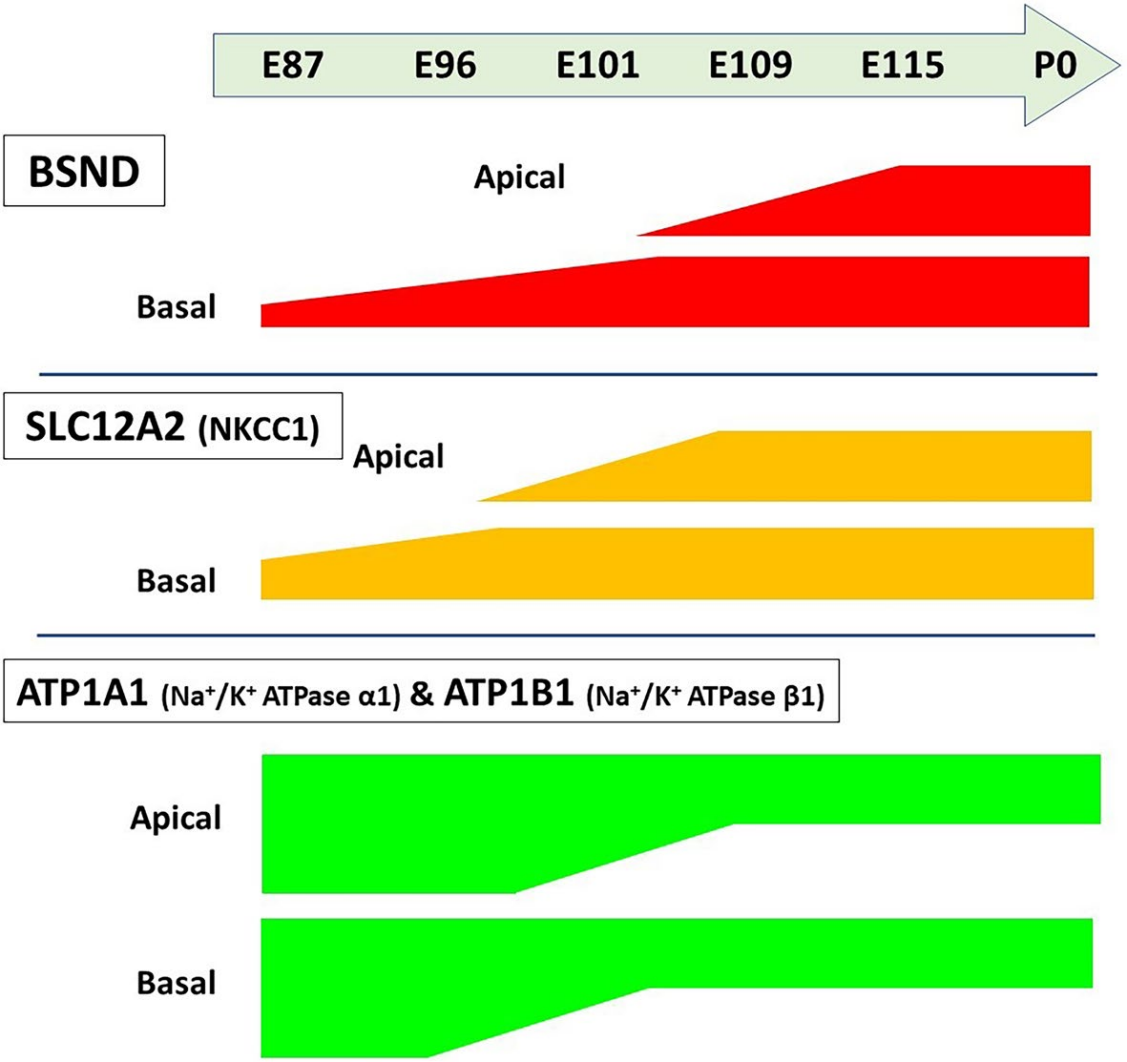
Schematic diagram depicting the expression patterns of specific markers in the stria vascularis marginal cells. BSND, SLC12A2, ATP1A1, and ATP1B1 are all marginal cell markers. However, during development, they have specific expression patterns in the cochlea. The expression of SLC12A2 in marginal cells precedes the expression of BSND in developing marginal cells. ATP1A1 and ATP1B1 show similar expression patterns; their expression was first ubiquitously observed in the cochlear epithelium and restricted to marginal cells in later steps.

Subsequently, we examined another subunit of Na^+^/K^+^ ATPase, ATP1B1, which was reportedly expressed in marginal cells of the stria vascularis^21^. Our observations revealed that expression patterns of ATP1B1 in marginal cells are similar to those of ATP1A1 (Fig. 3). At E87, ATP1B1 was ubiquitously expressed in the cochlear epithelium (Fig. 3A). After E96, expression was observed to be restricted to marginal cells (Fig. 3B–H). A schematic of the expression patterns of BSND, SLC12A2, ATP1A1, and ATP1B1 is depicted in Fig. 4.

### Integration and development of the intermediate cells of the stria vascularis in the primate

**Figure 5:**
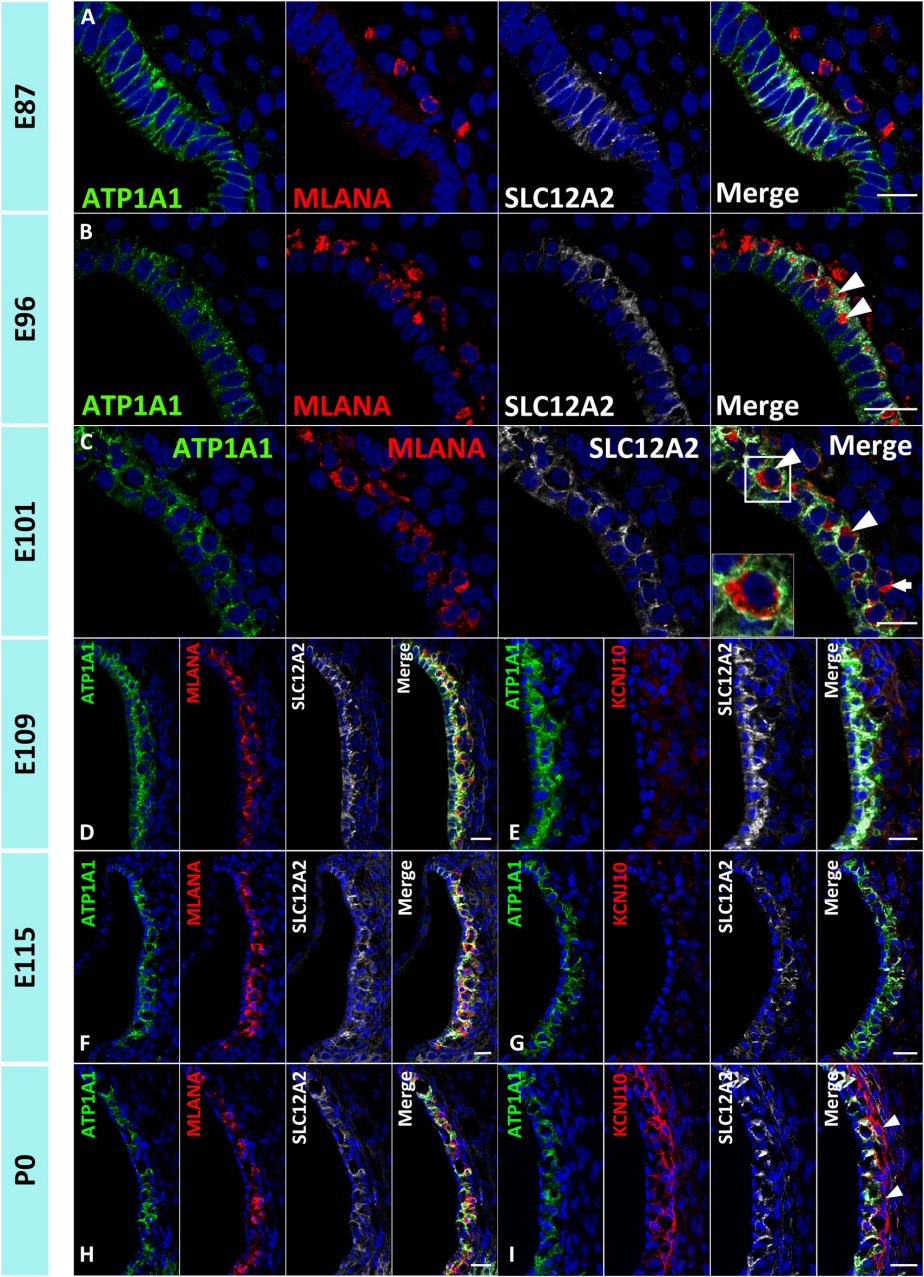
MLANA expression patterns and intermediate cell development in the stria vascularis. (**A**) In the E87 cochlea, a small number of MLANA-positive cells are observed next to SLC12A2-positive marginal cells. (**B**) In the E96 cochlea, an increase in MLANA-positive cells is observed, and several cells integrate into the marginal cell layers (Arrowheads in **B**). (**C**) In E101, most cells have integrated into the marginal cells (Arrowheads in **C**), whereas several cells have not (Arrow in **C**). An inset shows a higher magnification image of the box. (**D** and **E**) At E109, intermediate cells are being complicated in the basal membrane of marginal cells. No KCNJ10 expression is detected at this stage. (**F**–**I**) At E115 and P0, MLANA-positive intermediate cells are morphologically similar to E109. While no KCNJ10 expression is detected at E115, KCNJ10 expression is detected in intermediate cells at P0. At P0, KCNJ10 expression is also detected in basal cells (Arrowheads in **I**). The nuclei were counterstained with Hoechst (blue). Scale bar: 20 µm.

Next, we examined the integration and development of intermediate cells of the stria vascularis by evaluating a marker of intermediate cells: melan-A (MLANA). The intermediate cells of the stria vascularis are melanocyte-like cells that originate from the neural crest cells and Schwann cell precursors, which immigrate into the cochlea, integrate into the stria vascularis, and, finally, develop into intermediate cells^14,15,18,19,28^. *MLANA* encodes a surface protein of melanocytes, Melan-A, whose expression is reported in developing intermediate cells^2^. We have previously reported MLANA-positive cells in the cochlea of the common marmoset, but a detailed examination was not performed^4^. In this study, we analyzed the immigration and integration of the MLANA positive cell in detail (Fig. 5). In addition, we examined the expression of KCNJ10 as a second marker for intermediate cells. *KCNJ10* encodes a KCNJ10 (Potassium Inwardly Rectifying Channel Subfamily J Member 10), which is also known as Kir 4.1. Expression of KCNJ10 is reported in the intermediate cells^29^.

In E87 cochlea, a small number of MLANA-positive cells were observed next to SLC12A2-positive marginal cells, and no MLANA-positive cells had integrated into marginal cell layers (Fig. 5A). However, an increase in MLANA-positive cells was observed in the E96 cochlea, and several cells were integrated into the marginal cell layers (Fig. 5B). At E101, most MLANA-positive cells were integrated into marginal cells (Fig. 5C). After E109, entirely developed intermediate cells were being complicated in the basal membrane of marginal cells (Fig. 5D–I). While KCNJ10 expression in the intermediate cells was not observed at E109 and E115, KCNJ10 expression in the intermediate cells was observed at P0.

### Capillary development of the stria vascularis in the primate

**Figure 6:**
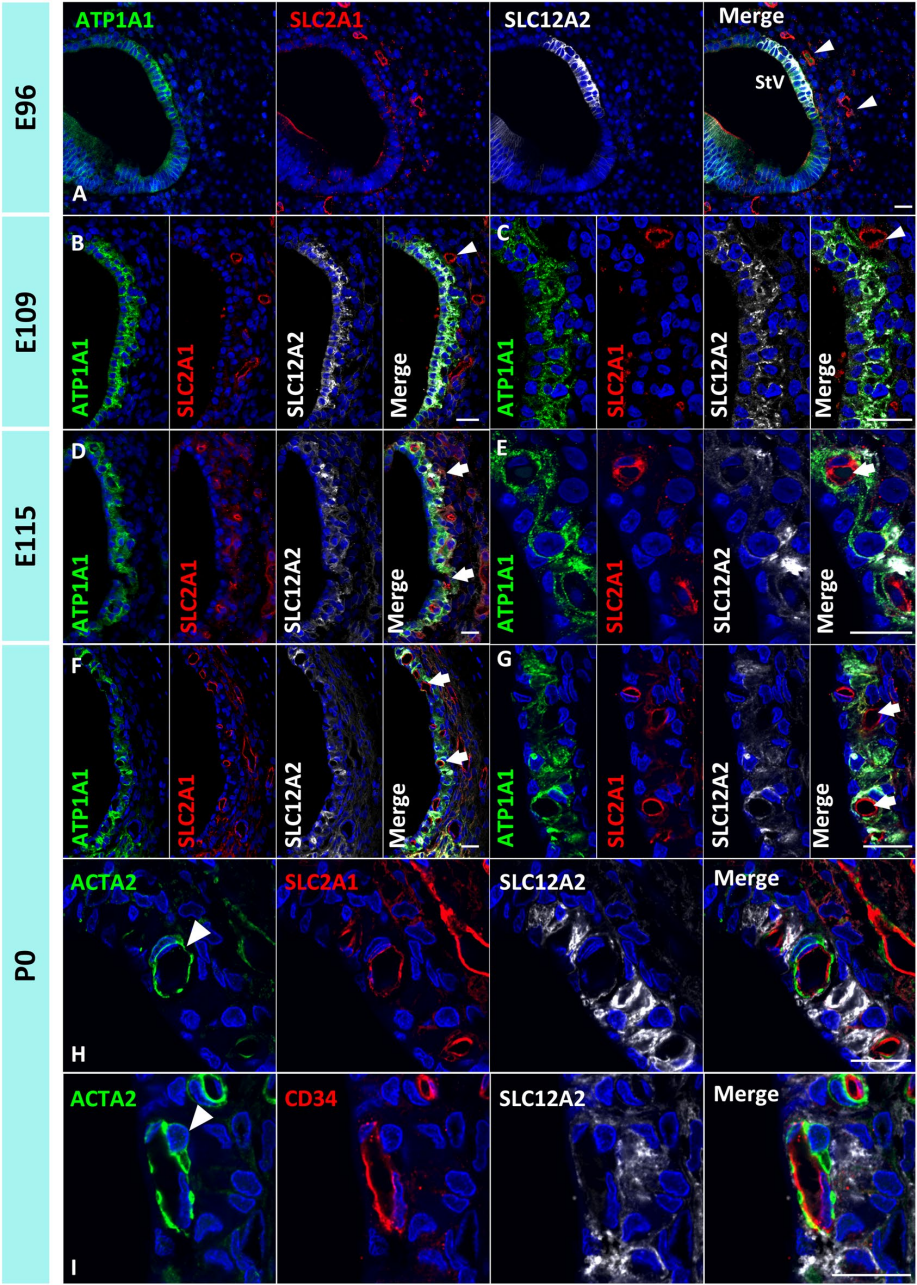
Capillary integration in the stria vascularis of the primate cochlea. (**A**) In the E96 cochlea, capillaries are not observed in the region next to SLC12A2-positive developing marginal cells (Arrowheads in **A**). (**B** and **C**) In E109, SLC2A1-positive capillaries are observed next to the marginal cell, whereas marginal cells cannot envelop the capillaries (Arrowheads in **B**). (**D** and **E**) At E115, enveloped capillaries are observed in the stria vascularis (Arrows in **D** and **E**). (**F**–**H**) At P0, enveloped capillaries are observed in the stria vascularis (Arrows in **F** and **G**). These SLC2A1 and CD34-positive capillaries are enveloped by ACTA2-positive pericytes (Arrowheads in **H** and **I**). The nuclei were counterstained with Hoechst (blue). Scale bar: 20 µm.

In mature stria vascularis, the numerous intraepithelial capillaries enveloped by pericytes are present^30,31^. *SLC2A1* encodes glucose transporter 1 (GLUT1), which is reportedly in the endothelial cells of the capillaries of the stria vascularis^2,32,33^. It is also known that the endothelial cells show CD34 expression^34^. A previous study showed the intraepithelial capillaries are enveloped by ACTA2 (Actin Alpha 2 Smooth Muscle, α-SMA)-positive pericytes in humans^35^. In this study, in addition to the marginal, intermediate, and basal cells, we also investigated the formation of the capillaries of the stria vascularis during cochlear development (Fig. 6).

In the E96 cochlea, capillaries were not observed in the region adjacent to the SLC12A2-positive developing marginal cells (Fig. 6A). At E109, SLC2A1-positive capillaries could be observed adjacent to the marginal cells, whereas marginal cells had not integrated the capillaries (Fig. 6B,C). After E115, enveloped capillaries were observed in the stria vascularis (Fig. 6D–G). These SLC2A1-positive capillaries, which are also CD34-positive, were enveloped by ACTA2-positive pericytes (Fig. 6H,I).

### Development of the basal cells of the stria vascularis in the primate

**Figure 7:**
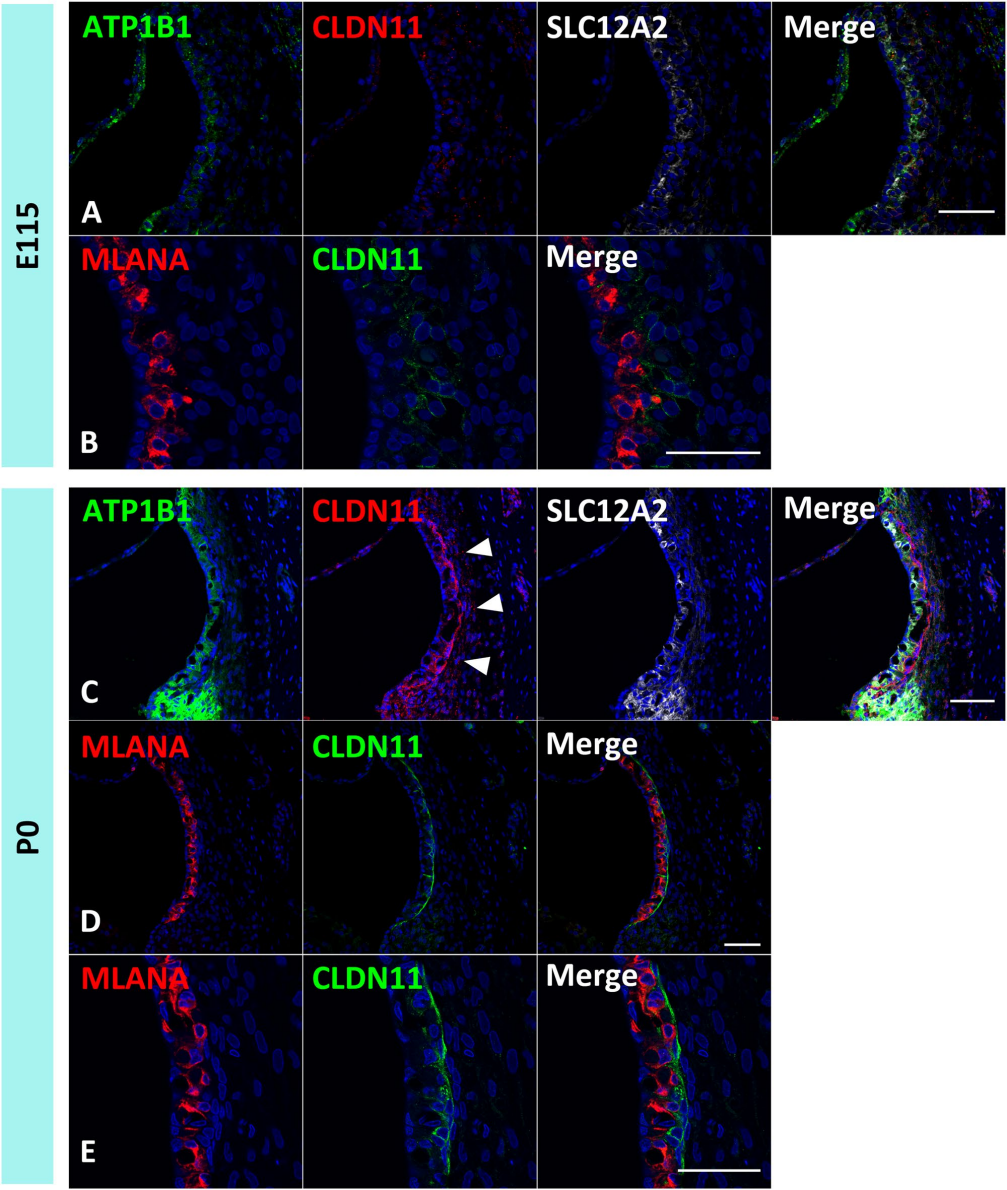
CLDN11 expression patterns and basal cell development in the stria vascularis. (**A** and **B**) In the E115 cochlea, only a slight expression of CLDN11 is observed, and no characteristic lining expression of CLDN11 in the basal cells is detected. (**C**–**E**) In the P0 cochlear, lining expression of CLDN11 along with the stria vascular has been detected (Arrowheads in **C**). The nuclei were counterstained with Hoechst (blue). Scale bar: 50 µm.

Finally, we examined the development of the basal cells of the stria vascularis by evaluating a marker of the basal cells, CLDN11 (Fig. 7). *CLDN11* encodes CLDN11, which belongs to the claudin family of tight junction-associated proteins and is a major component of myelin, a vital component of the central nervous system^36^. CLDN11 expression in basal cells has been observed in rodents^37^ and humans^38^. The expression of this protein in the basal cells of the stria vascularis is essential for generating endocochlear potential in the cochlea and normal hearing^37^.

At E115, we observed only a slight expression of CLDN11, and no characteristic lining expression of CLDN11 in the basal cells was detected (Fig. 7A,B). At P0 cochlea, CLDN11 expression was observed in the basal cells lining the basal side of stria vascularis (Fig. 7C–E). This observation suggested that the functional maturation of basal cells is completed in the late embryonic stages in this primate, at least after E115.

## Discussion

**Figure 8:**
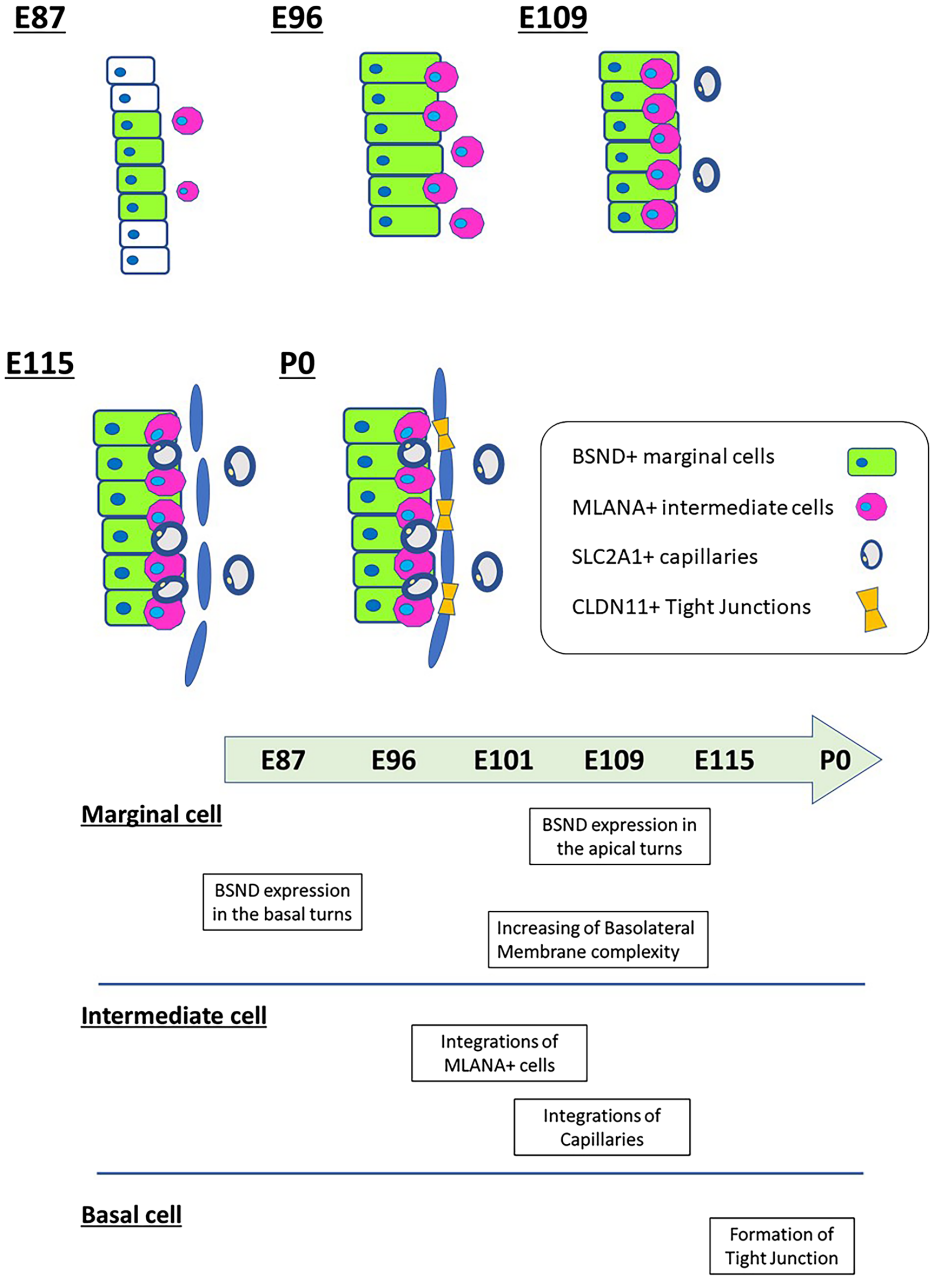
Schematic of the development of the stria vascularis of the common marmoset. Specification and maturation of marginal cells are followed by intermediate cells and capillary integration. The formation of tight junctions in the basal cell, which is essential for generating endocochlear potential, is observed in the late embryonic stages.

In this study, we have described the development of the stria vascularis in a primate animal model for the first time. We observed that in the cochlea of this primate, the development of the stria vascularis begins with the specification of the marginal cells, followed by the integration of intermediate cells derived from migrating neural crest cells, as previously reported in rodents^13^ and humans^2,3^. The development of the stria vascularis is accomplished by the differentiation of the basal cells after the capillary intrusion. A schematic of the stria vascularis in this primate is shown in Fig. 8. The development of the stria vascularis in several species, including rodents (20–22) and humans^2,3,17^, was previously reported. Similar to the development reported in both mice^13^ and humans^3^, the development of basal turns of the stria vascularis precedes the development of apical turns in the common marmoset.

In the human fetus, marginal cells of the stria vascularis are thought to appear at 11 weeks of gestation (GW). By 10 GW, future stria vascularis can be distinguished only by their location on the lateral wall of the cochlear duct^3^. At 12–13 GW, the cellular process of marginal cells develops intensively in intermediate cells^3^. Although the first feature of basal cells is observed at 14 GW, tight junction formation in basal cells is only observed after 20 GW. A more recent observation of gene expression patterns in human fetuses has offered further insights into the development of the stria vascularis^2^. ATP1A1 expression was observed at 10.4 GW in the entire epithelium of the cochlear duct, whereas ATP1A1 expression was gradually restricted to the basolateral membrane of marginal cells by 18GW. MLANA-positive cell invasion in marginal cells was observed at basal turns from 12GW, and integration into marginal cells was reported at 16GW. At 18GW, MLANA-positive cells were tightly integrated into the marginal cells^2^. Expression of KCNJ10 in intermediate cells was not observed in 18GW^2^. While comparing sensory epithelium development between the two species, we found that the E87 cochlea in common marmosets corresponds to about 12 GW in humans and the E115 cochlea in common marmosets to 20 GW in humans^4,6^. These reports indicate that stria vascularis development is largely well-preserved between humans and primates and is slower than in rodents^13^.

However, the point at which development involves the appearance of an endocochlear potential in the common marmoset fetus remains unknown. In prior studies on rodents, endocochlear potential has been observed immediately before the onset of hearing and coincides with the morphological maturation of gap and tight junctions^15,39^. Based on the observations that tight junction formation in basal cells of the stria vascularis becomes evident and gap junctions in lateral wall fibrocytes mature after E115^40^, we suggest that endocochlear potential and hearing in the marmoset fetus do not emerge before E115. This argument is similar to the supposition that human endocochlear potential does not emerge before the third trimester of pregnancy^2^. Our speculation on the timing of formation of endocochlear potential based on our histological observations could be helpful in future studies that measure endocochlear potential in the fetuses of this primate.

This study is the first to investigate, sequentially and in detail, the development of the stria vascularis in a non-human primate fetus alongside its molecular expression patterns (37, 38). We have described the development of the stria vascularis in the common marmoset by examining the localization of critical molecules using immunohistochemistry. Our results indicate that the development of the stria vascularis in this primate is similar to that in humans. Therefore, this model animal could be a substitute for the human fetus, which is difficult to study because of ethical challenges.

Researchers have demonstrated the involvement of the stria vascularis in drug-induced or age-related hearing loss^41–43^. To overcome this damage, regenerative therapy of the stria vascularis would be a feasible approach^11^. Studies on regenerative therapy would benefit from the information on the development of the stria vascularis in a primate model.

Gestation periods and developmental speeds can vary widely between species. In general, body pattern formation in an embryo, a process known as segmentation, progresses more slowly in humans than in mice^44,45^. Compared to rodents, cochlear development in primates takes more than three times longer^1,4,46,47^. Such long periods of development help reveal transient changes in the expression of genes. For example, transient expression of SLC12A2 in the organ of Corti^4^ and developmental changes of the synaptotagmins in outer hair cells have been discovered in primates^5^ but not in rodents. In this study, we highlighted the gaps in the initial expression timing of BSND and SLC12A2; BSND expression follows SLC12A2 expression. While these differences can be attributed to a disparity in the speed of protein synthesis in these genes, both genes may also be independently controlled, suggesting that specification in marginal cells is more complex than was initially assumed. Studies are warranted to further investigate this possibility.

The limitations of this study include that we examined the expression patterns of a part of the marker proteins of the stria vascularis. While expression patterns of these proteins observed in this study would be useful for understanding the general developmental time course of the stria vascularis in this primate model animal, it would be necessary to conduct more detailed examinations of gene expression patterns and functional assays of cells in the stria vascularis in this non-human primate model animal in the future.

## Conclusions

In conclusion, in this study, we examined the development of the stria vascularis in the cochlea of the primate animal model. We revealed the basic time course of the development of the stria vascularis for cell compartments in marginal cells, intermediate cells, and basal cells. The study findings will be valuable in future developmental studies on the primate cochlea, as well as for studies on regenerative therapy of the stria vascularis.

## Data Availability

All data generated or analyzed during this study are included in this published article.

## Abbreviations

ATP1A1: ATPase Na^+^/K^+^ transporting subunit alpha 1
BSND: Barttin CLCNK type accessory subunit beta
CLC: Chloride channels
CLDN11: Claudin 11
KCNJ10: Potassium inwardly rectifying channel subfamily J member 10
GLUT1: Glucose transporter 1
ACTA2: Actin alpha 2 smooth suscle
GW: Weeks of gestation
MLANA: Melan-A
NKCC1: Na^+^/K^+^/2Cl^−^ co-transporter
SLC12A2: Solute carrier family 12 member 2
